# A Young Woman with Common Variable Immunodeficiency: The Role of Thorough Medical History and Physical Examination in Accurate Diagnosis

**DOI:** 10.1155/2024/4028888

**Published:** 2024-05-10

**Authors:** Amirhossein Khodadadi, Rozita Khodashahi

**Affiliations:** ^1^School of Medicine, Mashhad University of Medical Sciences, Mashhad, Iran; ^2^Transplant Research Center, Clinical Research Institute, Mashhad University of Medical Sciences, Mashhad, Iran

## Abstract

Common variable immunodeficiency (CVID) is a rare immunodeficiency syndrome which presents with wide manifestations leading to delayed diagnosis. A 34-year-old woman presented to our hospital complaining of dyspnea and productive cough. Lung CT scan revealed loculated right-sided pleural effusion with bronchiectasis and consolidation in right lower lobes. After taking medical history and physical examination, we suspected CVID and ordered serum immunoglobulin levels. The laboratory results were in line with CVID diagnosis and showed decreased levels of IgG, IgM, and IgA. The patient was started on intravenous immune globulin (IVIG) therapy every month. After 3-month follow-up, the patient reported no problem and felt better.

## 1. Introduction

Common variable immunodeficiency (CVID) is the most common symptomatic primary immunodeficiency in adults with defective immunoglobulin production [1, 2]. CVID has a wide range of presentations, including recurrent bacterial infections, autoimmune disorders, allergic disorders, inflammatory disorders, and malignancies [3]. These wide presentations cause patients to be evaluated by multiple specialists before they are diagnosed; hence, delayed diagnosis is prevalent. CVID is commonly diagnosed between the ages of 20 and 45 [3, 4].

The Middle East and North Africa (MENA) region has high rate of inborn errors of immunity (IEI) particularly due to high frequency of consanguineous marriages. This trend yields to a high rate of autosomal recessive forms of IEI [5, 6]. CVID belongs to IEI entity and is the second most common IEI in MENA region [5]. MENA region is the origin of 33.1% of 485 described genes related to IEI and IEI has the estimated prevalence of 2.96 in 100,000 in MENA which is higher than any region worldwide [6].

The diagnosis of CVID relies on reduced serum levels of IgG, IgA, and IgM [3, 6, 7]. After making diagnosis, the mainstay of treatment is administration of intravenous immune globulin (IVIG) every three to four weeks either intravenously or subcutaneously [6, 8, 9].

In this paper, we present a 34-year-old woman diagnosed with CVID in Mashhad, Iran.

## 2. Case Presentation

**Figure 1:**
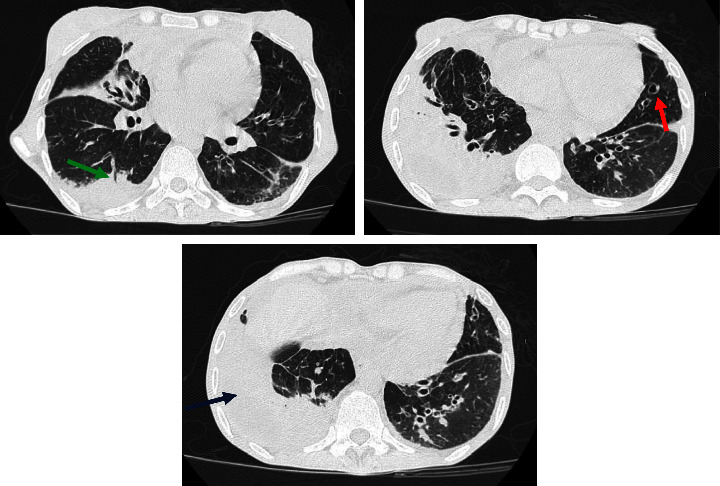
Three different sections of lung CT scan of the patient at admission time showing pleural effusion (blue arrow), consolidation (green arrow), and bronchiectasis (red arrow).

A 34-year-old woman presented to Imam Reza Hospital with a 1-month history of dyspnea, productive cough, and rhinorrhea in December 2023. She was hospitalized in other center for two weeks with the diagnosis of COVID-19 but she did not feel better, so she decided to attend our center. In emergency department, she underwent lung CT scan which revealed loculated right-sided pleural effusion with consolidation which was dominant in right lower lobes (Figure 1). Bronchiectasis was present in both lung fields (Figure 1). Chest tube was placed and then she was admitted into infectious diseases ward with the diagnosis of bacterial pneumonia and concurrent empyema. After conducting thorough medical history and physical examination, some key findings were discovered which are as follows: two histories of bacterial meningitis in April 2022 and April 2023 leading to ICU admission, chronic cough, chronic night sweating, recurrent common cold episodes, splenomegaly, secondary amenorrhea, and cachexia.

The onset of her medical issues was 9 years earlier when she developed chronic nonproductive cough. Owing to living in an endemic area, tuberculosis was ruled out. Then, she received salbutamol and ipratropium bromide spray for her cough without any specific diagnosis.

Seven years earlier, during her ultrasonography for second pregnancy, she was incidentally diagnosed with splenomegaly and referred to a hematologist. Furthermore, she also had anemia and thrombocytopenia in her pregnancy follow-up visits. She underwent bone marrow biopsy which was normal. She reported that she received a diagnosis of immune thrombocytopenic purpura (ITP) and was candidate for splenectomy which was not performed. After giving birth to her second child, she developed secondary amenorrhea and significant weight loss. During the preceding 7 years, she experienced recurrent common cold episodes at least once a month, one episode of acute unilateral otitis media, and two episodes of bacterial meningitis.

Putting together these notable findings, we suspect CVID and human immunodeficiency virus (HIV) as the cause of our patient's presentation. The laboratory results are shown in Table 1. The immunoglobulin assay revealed decreased levels of IgG, IgM, and IgA which were consistent with CVID diagnosis.

We started IVIG therapy with the dose of 600 mg/kg and the patient was discharged one week later with the prescription of IVIG infusion every month. After 3-month follow-up, she was in a good general health and reported no problem.

## 3. Discussion

CVID is a rare immunodeficiency syndrome with an incidence of 1 in 25,000 to 1 in 100,000. It is usually diagnosed between the ages of 20 and 45 [3, 4]. Our patient was also diagnosed at this age range. There was a 9-year delay in our patient diagnosis, slightly higher than previous studies [3].

Approximately 61% of IEI in MENA region has consanguinity [5]; however, our patient was not born from consanguineous marriage.

CVID presents with variable and wide manifestations. In a study by Gathmann et al. [4], 26% and 23% of patients presented with splenomegaly and bronchiectasis, respectively. Similarly, our patient also had splenomegaly and bronchiectasis. The most common presenting symptom is infection, with sinopulmonary being the most common infection [4, 10, 11]. In current admission, our patient had pneumonia with empyema and she also had one history of acute otitis media. Collectively, these infections are considered sinopulmonary infections. Our patient also had 2 episodes of bacterial meningitis which is not common according to previous studies [4]. In a study by Zhou et al. [12], their patient had secondary amenorrhea like our patient. To the best of our knowledge, there is only one report of secondary amenorrhea in CVID patients in the literature in addition to our patient.

Gastrointestinal symptoms are common in CVID [10, 13], although our patient did not complain of them. However, her weight loss can be related to her insidious malabsorption.

Patients with unknown CVID are frequently referred to hematologists due to disturbances in the blood cell counts (cytopenias), splenomegaly, and lymphadenopathy [3, 14]. Similarly, our patient was also referred to a hematologist and received the diagnosis of ITP. ITP is among the most common autoimmune cytopenias in CVID [3, 15].

Overall, we should consider CVID in patients with multiple infections and seemingly different unrelated symptoms [6].

## 4. Conclusion

The case presented here highlights the importance of history taking and physical examination to diagnose immunodeficiency syndromes like CVID. The patient herself or himself tells us the diagnosis if we listen to them and consider IEI diagnosis guidelines. Then, we can start treatment promptly and reduce morbidity and mortality in the patients.

## Figures and Tables

**Table 1:** Laboratory test results.

Laboratory test	Result	Range
IgG (mg/dL)	45	700–1600
IgA (mg/dL)	5	70–400
IgM (mg/dL)	25	40–230
HIV antibody	0.13	Negative <1 Positive >1
Antinuclear antibody (ANA)	0.14	Negative <1 Positive ≥1

## Data Availability

Access to data is permitted with the authors' permission.

## Abbreviations

CVID: Common variable immunodeficiency
IVIG: Intravenous immunoglobulin
MENA: Middle East and North Africa
IEI: Inborn errors of immunity
ITP: Immune thrombocytopenic purpura
HIV: Human immunodeficiency virus.
